# Clinical Characteristics of Patients With Dizziness/Vertigo Accompanied by Posterior Semicircular Canal Hypofunction

**DOI:** 10.3389/fmed.2021.589969

**Published:** 2021-04-13

**Authors:** Zheyuan Li, Bo Liu, Hongli Si, Kangzhi Li, Bo Shen, Xiang Li, Xia Ling, Xu Yang

**Affiliations:** ^1^Peking University Aerospace School of Clinical Medicine, Beijing, China; ^2^The First Affiliated Hospital of Jinzhou Medical University, Jinzhou, China; ^3^Department of Neurology, Aerospace Center Hospital, Peking University Aerospace School of Clinical Medicine, Beijing, China

**Keywords:** video-head-impulse test, loss of the posterior canal(s), labyrinthine infarction, vestibular neuritis, autoimmune inner ear disease, vestibular migraine

## Abstract

**Objective:** To investigate the clinical characteristics of patients with dizziness/vertigo accompanied by loss of the posterior canal(s) (LPC).

**Methods:** Clinical data of 23 patients with LPC were collected. We determined video-head-impulse test (vHIT) gains of all six semicircular canals and correlated vHIT findings with other vestibulo-cochlear tests, including caloric test, ocular and cervical vestibular-evoked myogenic potentials (oVEMP, cVEMP), pure tone audiometry (PTA), and analyzed the differences in clinical manifestations of patients with LPC with different etiologies.

**Results:** LPC was identified in 23 patients. At the time of disease onset, most patients presented with dizziness (47.8%) and vertigo (30.4%) only, and some patients (21.7%) complained of unsteadiness. Among these 23 patients with LPC, there were 14 (60.9%) patients of isolated LPC (ILPC), 21 (91.3%) patients of unilateral LPC (ULPC), and 2 (8.7%) patients of bilateral LPC (BLPC). (1) Among 14 patients with ILPC, 13 (92.9%) patients had unilateral ILPC, the rate of ipsilesional impairment on caloric test, or oVEMP/cVEMP test or PTA ipsilesionally was 53.8% (7/13) in patients with unilateral ILPC. The causes of unilateral ILPC were vertigo/dizziness of unclear origin (38.5%), labyrinthine infarction (15.4%), vestibular migraine (15.4%), and other diseases (30.8%); (2) among 21 patients with ULPC, 7 patients (33.3%) were accompanied with horizontal semicircular canal hypofunction ipsilesionally, the abnormal rate of caloric test, or oVEMP/cVEMP tests or PTA ipsilesionally was 57.1%. The causes of ULPC were vertigo/dizziness of unclear origin (33.3%), autoimmune inner ear disease (14.3%), labyrinthine infarction (14.3%), vestibular neuritis (9.5%), vestibular migraine (9.5%), and other diseases (19.0%); (3) among two patients with BLPC, one patient presented with unsteadiness, the causes of BLPC were vestibular paroxysmia and autoimmune inner ear disease.

**Conclusion:** vHIT is a fast and effective method for assessing LPC, which can be used to detect isolated PC dysfunction. The causes of ILPC were peripheral origin or central origin. Patients with ILPC and ULPC mostly presented with dizziness/vertigo, and ULPC was often accompanied by ipsilateral vestibulo-cochlear impairment.

## Introduction

Peripheral vestibular disease is the most common cause of vertigo. Clinical evaluation of peripheral vestibular function often relies on vestibulo-cochlear tests, such as caloric irrigation, vestibular-evoked myogenic potential (VEMP) test and pure tone audiometry (PTA) (1), however, these tests cannot evaluate the function of the posterior semicircular canal(s) (PSCC). With the introduction of video-head-impulse test (vHIT), it is now possible to detect the function of vertical semicircular canals (SCCs). Due to the lack of diagnostic methods and the anatomical proximity of PSCC (2, 3) and based on clinical experience, the loss of the posterior canal (s) (LPC) is clinically uncommon. In terms of anatomy, PSCC is indirectly connected to the inferior vestibular nerve and its accompanying blood vessels, the space that the inferior vestibular nerve travels through is relatively broader than the vestibular nerve trunk, as well as the superior vestibular nerve and its accompanying blood vessels. And the bony channel of the superior vestibular nerve is more than seven times longer than the bony channel of the inferior vestibular nerve, it is speculated that the superior vestibular nerve is more susceptible to vascular and inflammatory factors (4, 5), but the PSCC is innervated by the inferior vestibular nerve, so LPC is clinically rare. The clinical manifestations are extremely heterogeneous across patients with LPC, LPC can be accompanied by spontaneous, downbeat nystagmus in positional, or head-shaking test. Diagnosing LPC may be extremely difficult because there are no other characteristic symptoms except dizziness or vertigo (6). Therefore, in order to diagnose and treat patients more effectively, the clinical characteristics and possible etiology in patients with LPC need to be further clarified.

Here we included patients with dizziness/vertigo who were diagnosed with LPC by vHIT, collected the clinical baseline data, results of videonystagmograph, caloric irrigation, ocular and cervical VEMP (oVEMP, cVEMP), and PTA, evaluated the vestibulo-cochlear function of these patients and explored the possible etiology of LPC, with hope of improving diagnosis and treatment for LPC.

## Subjects and Methods

### Subjects

A total of 448 patients with dizziness/vertigo who received treatment in Vertigo center of Neurology department, Peking University Aerospace School of Clinical Medicine from December 2017 to 2019 were continuously enrolled. Twenty-three patients with LPC (including 13 patients with ILPC, eight patients with non-ILPC(LPC+HPC) and two patients with BLPC) were identified. Inclusion criteria were: (1) patients who complained of dizziness, vertigo, gait unsteadiness or hearing loss; (2) vHIT demonstrated reduced gain of <0.70 for unilateral or bilateral PSCC (at least 20 head impulses for each canal) (7). Exclusion criteria were: (1) vHIT demonstrated reduced gain of <0.70 for all three semicircular canals including ASCC in ULPC and BLPC since all three canal impairments correspond to unilateral or bilateral vestibulopathy rather than ILPC; (2) patients who had central nervous system diseases, and those who had neck disorders, and did not cooperate well with the examination. This study was approved by the ethics committee of Aerospace Center Hospital, Peking University Aerospace School of Clinical Medicine, Beijing, China. Written informed consent was obtained from all patients.

### Clinical Data Collection

**Figure 1:**
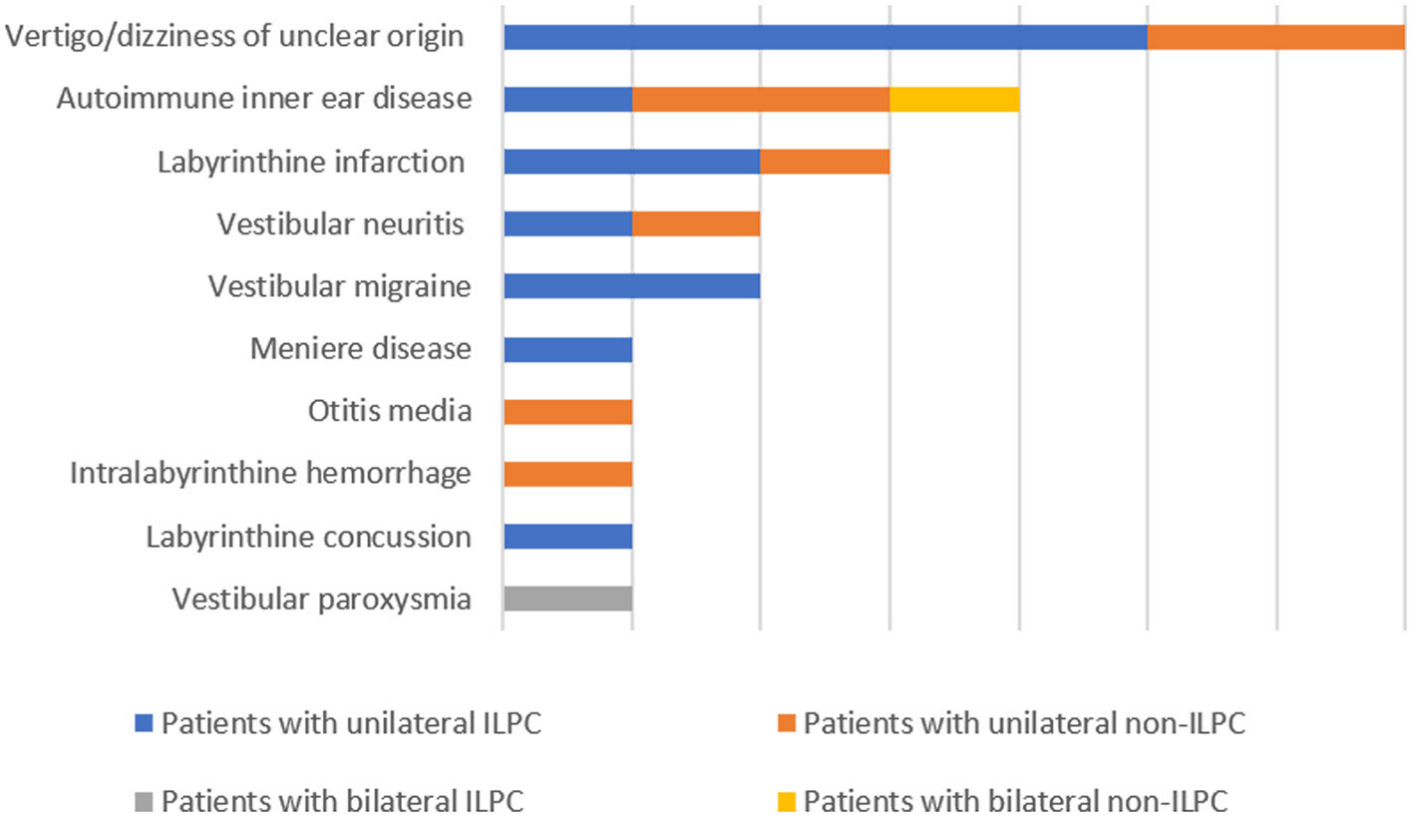
Different diagnosis in patients with the loss of the posterior canal (s) (LPC).

The following data were collected from patients' medical records: sex, age, current medical history (course of disease, symptoms during attack, duration of attack, factors induced the attacks, triggering factors, and accompanying symptoms), underlying diagnosis (including labyrinthine infarction, dizziness/vertigo of unclear origin, vestibular neuritis, autoimmune inner ear disease, Ménière's disease, vestibular migraine, otitis media, vestibular paroxysmia, labyrinthine concussion, intralabyrinthine hemorrhage) (Figure 1), results of physical examination upon admission, and auxiliary examinations (such as immune-related laboratory tests, MRI and CT of the brain). Regarding the diagnosis of the above-mentioned underlying diagnosis, labyrinthine infarction, vestibular neuritis, labyrinthine concussion and intralabyrinthine hemorrhage are diagnosed according to patients' medical history, cochleo-vestibular evaluation and neurovascular imaging (8–11), vestibular migraine, Ménière's disease and vestibular paroxysmia are diagnosed based on the diagnostic criteria suggested by Barany society (12–14), autoimmune inner ear disease is diagnosed according the medical history, vestibulocochlear evaluation and immune-related laboratory tests (15, 16), otitis media is diagnosed according to the criteria reported by Gates et al. (17).

### Vestibulo-Cochlear Tests

vHIT (Interacoustics, Middelfart, Denmark) was used to assess the function of all the six SCCs by measuring the gain of the vestibulo-ocular reflex. The instrument comprises an inertial measurement unit to measure movements of the head and an infrared camera to record eye movements. vHIT can calculate the mean gain value (ratio of eye to head velocity) for each of the six SCCs, and also detect covert or overt saccades. During the test, goggles were secured firmly to patients' head to ensure that the goggles did not slip from the face during head movement. Patients sat ~1.5 m in front of a wall on which a visual target was affixed. Before testing, head movement and eye movement were calibrated according to the manufacturer's instructions. Patients were told about the detailed test process to ensure that the patient can cooperate with the examination, and patients were asked to sit in upright posture and keep their eyes fixed on the target on the wall while the examiner applies passive, unpredictable, low amplitude and high acceleration head impulses in the planes of stimulation (horizontal plane, right-anterior-left-posterior plane, left-anterior-right-posterior plane) (18). At least 20 acceptable head impulses for each SCC were required. According to manufacture recommendations, normal gain values are expected to range between 0.80 and 1.20 for horizontal canals (19, 20), and 0.70–1.20 for vertical canals (7). Pathological saccades and gain values below the normal range were recorded.

Video-nystagmography (VNG) (Interacoustics, Middelfart, Denmark) was used to measure and record patients' eye movement, including spontaneous nystagmus (SN), gaze-evoked nystagmus, saccades, smooth pursuit, optokinetic, and head-shaking nystagmus (HSN) (21). During the caloric test, canal paresis (CP) value > 25% was defined as unilateral horizontal SCC (HSCC) dysfunction (22). The sum of the maximal peak velocities of the slow phase of the induced nystagmus for warm and cold water stimulation on each side <6°/s was defined as bilateral HSCC dysfunction (23).

In order to assess the function of the otolithic organs, air conduction oVEMP (Interacoustics, Middelfart, Denmark) was used to assess utricle and superior vestibular nerve function (24), and air conduction cVEMP (Interacoustics, Middelfart, Denmark) was used to assess saccular and inferior vestibular nerve function (25).

In addition, all patients underwent PTA, hearing loss at six frequencies (250 Hz, 500 Hz, 1 kHz, 2 kHz, 4 k Hz, and 8 kHz) was determined.

### Statistical Analysis

All data are expressed as the mean ± SD. Enumeration data are expressed as percentage. The chi-square test was used to compare count data between groups. Yates's correction for continuity or the Fisher exact test was used if necessary. All data were analyzed by a two-tailed test, and a level of *P* < 0.05 was considered statistically significant. All the analyses were performed using SPSS 22.0 software.

## Results

### Clinical Baseline Characteristics of Patients

A total of 448 patients with dizziness/vertigo were continuously enrolled in the study, 23/448 (5.1%) patients were diagnosed with LPC using vHIT. Ten (43.5%) patients were males. The mean age of patients was 57.0 ± 14.6 years (range 20–82 years). There were 21 (91.3%) patients with ULPC and 2 (8.7%) patients with BLPC.

Vertigo was presented in 8 (34.8%) patients, including seven patients with ULPC, and 1 patient with BLPC, dizziness was presented in 15 (65.2%) patients, including 14 patients with ULPC, and one patient with BLPC. And among all patients with LPC, 5 (21.7 %) patients presented with gait unsteadiness, including four patients with ULPC, and one patient with BLPC.

**Figure 2:**
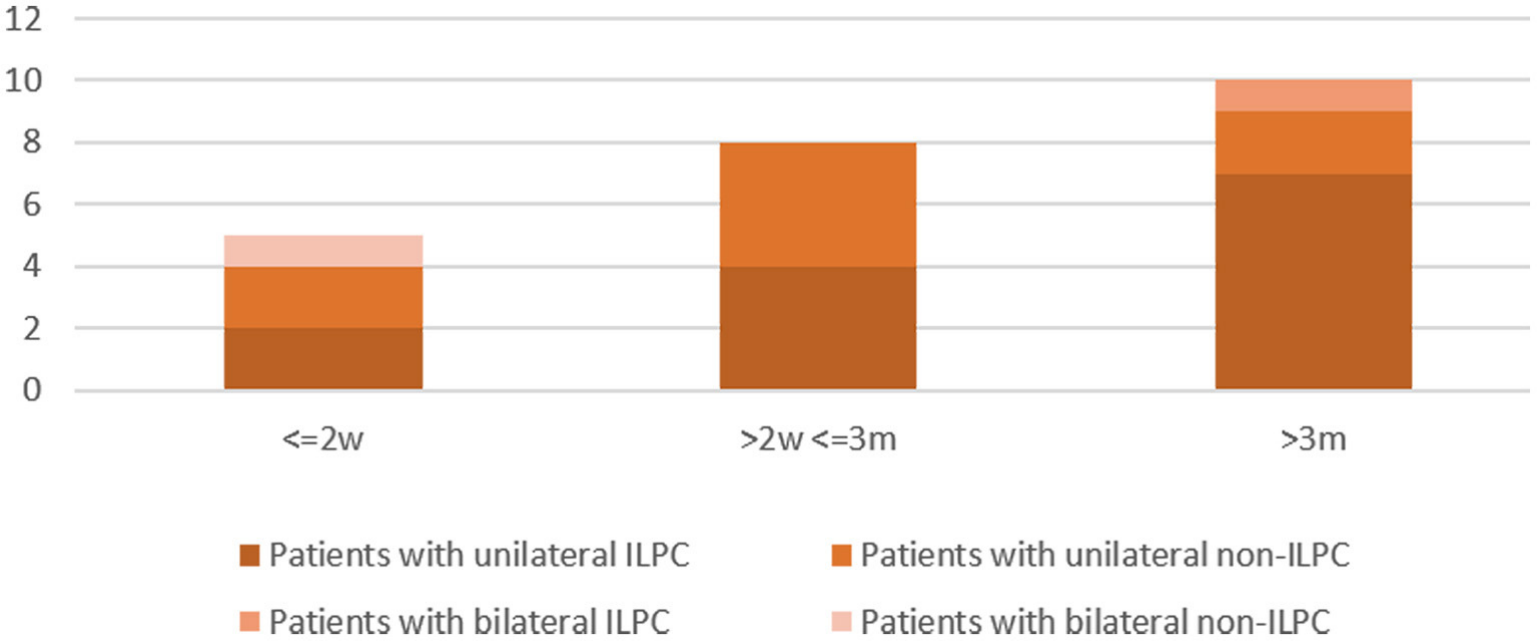
Course of disease in patients with the loss of the posterior canal (s) (LPC).

Disease duration was 0–2 weeks in 5 (21.7%) patients (unilateral ILPC = 2, unilateral non-ILPC = 2, bilateral non-ILPC = 1), 2 weeks-3 months in 8 (34.8%) patients (unilateral ILPC = 4, unilateral non-ILPC = 4), > 3 months in 10 (43.5%) patients (unilateral ILPC = 7, unilateral non-ILPC = 2, bilateral ILPC = 1) (Figure 2).

The underlying diagnoses were labyrinthine infarction in 3 (13.0%) patients (ILPC = 2, non-ILPC = 1), vertigo/dizziness of unclear origin in 7(30.4%) patients (ILPC = 5, non-ILPC = 2), vestibular neuritis in 2 (8.7%) patients (ILPC = 1, non-ILPC = 1), autoimmune inner ear disease in 4 (17.4%) patients (ILPC = 1, non-ILPC = 3), vestibular migraine in 2 (8.7%) patients (ILPC = 2, non-ILPC = 0), otitis media in 1 (4.3%) patient (ILPC = 0, non-ILPC = 1), Ménière's disease in 1 (4.3%) patient (ILPC = 1, non-ILPC = 0), vestibular paroxysmia in 1 (4.3%) patient (ILPC = 1, non-ILPC = 0), labyrinthine concussion in 1 (4.3%) patient (ILPC = 1, non-ILPC = 0), intralabyrinthine hemorrhage in 1 (4.3%) patient (ILPC = 0, non-ILPC = 1) (Tables 1, **5**, Figure 1).

**Table 1:** Underlying diagnoses in patients with LPC on vHIT.

	**Patients with unilateral LPC**		**Patients with bilateral LPC**		
**Diagnosis**	**ILPC**	**Non-ILPC**	**ILPC**	**Non-ILPC**	**Total (*n*/%)**
Vertigo/dizziness of unclear origin	5	2	0	0	7 (30.4)
Autoimmune inner ear disease	1	2	0	1	4 (17.4)
Labyrinthine infarction	2	1	0	0	3 (13.0)
Vestibular neuritis	1	1	0	0	2 (8.7)
Vestibular migraine	2	0	0	0	2 (8.7)
Meniere disease	1	0	0	0	1 (4.3)
Otitis media	0	1	0	0	1 (4.3)
Intralabyrinthine hemorrhage	0	1	0	0	1 (4.3)
Labyrinthine concussion	1	0	0	0	1 (4.3)
Vestibular paroxysmia	0	0	1	0	1 (4.3)
**Total**	21 (91.3)		2 (8.7)		23 (100)

*LPC, loss of the posterior canal(s); vHIT, video-head-impulse test*.

The past medical history included risk factors for atherosclerosis (including hypertension, hyperlipidemia, cigarette smoking and diabetes mellitus) (*n* = 7, 30.4%, unilateral ILPC = 4, unilateral non-ILPC = 3), benign paroxysmal positional vertigo (*n* = 5, 21.7%, unilateral ILPC = 4, unilateral non-ILPC = 0, bilateral non-ILPC = 1), autoimmune diseases (*n* = 4, 17.4%, unilateral ILPC = 1, unilateral non-ILPC = 3), migraine (*n* = 2, 8.7%, unilateral ILPC = 2, unilateral non-ILPC = 0), concussion (*n* = 1, 4.3%, unilateral ILPC = 1, unilateral non-ILPC = 0). And there were 4 (17.4%) patients (unilateral ILPC = 1, unilateral non-ILPC = 2, bilateral ILPC = 1) who had no specific past medical history.

### vHIT Results

ULPC was considered if vHIT showed gain reduction in unilateral PSCC with covert or overt saccades. Isolated LPC (ILPC) was considered if vHIT showed gain reduction only in unilateral or bilateral PSCC. Among 21 patients with ULPC, ILPC was confirmed in 13 (61.9%) patients, and the remaining 8 (38.1%) patients had HSCC hypofunction on vHIT, which occurred only ipsilesionally in 7 (7/8, 87.5%) patients, and another one patient had bilateral lesions. Among eight patients with HSCC hypofunction, no patients were accompanied with anterior SCC (ASCC) hypofunction on vHIT. Among different underlying diagnoses, rates of ipsilateral HSCC hypofunction on vHIT were different, being highest for vertigo/dizziness of unclear origin (5/7, 71.4%) and autoimmune inner ear disease (2/3, 66.7%) (Tables 2, **5**).

**Table 2:** Ipsilesional vestibulo-cochlear involvement in patients with unilateral LPC on vHIT.

**Diagnosis**	**vHIT: impaired horizontal canal (*n*, %)**	**Caloric irrigation^a^: impaired horizontal canal (*n*, %)**	**Impairment of any of the two: vHIT, Calorics (*n*, %)**	**oVEMPs^b^: impaired utriculus (*n*, %)**	**cVEMP^b^: impaired sacculus (*n*, %)**	**Impairment of any of the three: Calorics, oVEMP, cVEMP (*n*, %)**	**PTA (hearing loss^c^) (*n*, %)**	**Impairment of any of the four: Calorics, oVEMP, cVEMP, PTA (*n*, %)**
Vertigo/dizziness of unclear origin (*n* = 7)	2, 28.6%	4, 57.1%	4, 57.1%	1,14.3 %	1, 14.3%	4, 57.1%	1, 14.3%	5, 71.4%
Labyrinthine infarction (*n* = 3)	1, 33.3%	0, 0%	1, 33.3%	0, 0%	0, 0%	0, 0%	1, 33.3%	1, 33.3%
Autoimmune inner ear disease (*n* = 3)	1, 33.3%	1, 33.3%	1, 33.3%	0, 0%	0, 0%	1, 33.3%	1, 33.3%	2, 66.7%
Vestibular neuritis (*n* = 2)	1, 50%	1, 50%	2, 100%	0, 0% (1 missing)	0, 0% (1 missing)	1, 50%	0, 0%	1, 50%
Vestibular migraine (*n* = 2)	0, 0%	1, 50%	1, 50%	0, 0%	0, 0%	1, 50%	0, 0%	1, 50%
Various causes^d^ (*n* = 4)	2, 50%	1, 25%	2, 50%	1, 25%	0, 0%	2, 50%	1, 25%	2, 50%
Total unilateral LPC (*n* = 21)	7, 33.3%	8, 38.1%	11, 52.4%	2, 10% (1 missing)	1, 5% (1 missing)	9, 42.9%	4, 19%	12, 57.1%

a *Impairment of the horizontal canal was considered significant if the asymmetry ratio was >25% in favor of the other side*.

b *Impairment of vestibular-evoked myogenic potentials was considered significant if the asymmetry ratio was >30% in favor of the opposite side or if responses were bilaterally absent*.

c *Significant hearing-loss was defined as CPT-AMA values >20%*.

d *Various causes include: Meniere disease (n = 1), Otitis media (n = 1), Intralabyrinthine hemorrhage (n = 1), Labyrinthine concussion (n = 1)*.

*LPC, loss of the posterior canal(s); vHIT, video-head-impulse test; oVEMP, ocular vestibular-evoked myogenic potentials; cVEMP, cervical vestibular-evoked myogenic potential; PTA, pure tone audiometry*.

BLPC was considered if vHIT showed gain reduction and saccades in bilateral PSCC, with or without accompanying gain reduction and saccades in other canals. Among two patients with BLPC, ILPC was confirmed in one patient, and the remaining one patient was accompanied with HSCC hypofunction on vHIT (**Table 5**).

### Caloric Irrigation and Eye Movement Measurements Results

Among 21 patients with ULPC, a CP value of >25% was found in 9 (42.9%) patients. Of these nine patients, ipsilesional responses to caloric irrigation were impaired in 8 (88.9%) patients. Among different underlying diagnoses, the rates of ipsilesional impairment on caloric irrigation were different, being highest for vertigo/dizziness of unclear origin (4/7, 57.1%, Table 2). Among 13 patients with ILPC, CP value of >25% was found in 5 (38.5%) patients. And of these five patients, ipsilesional responses to caloric irrigation were impaired in 4 (80%) patients (Table 3). Among 21 patients with ULPC, SN was found in seven patients (2 patients with ipsilesional beating nystagmus, three patients with contralesional beating nystagmus, one patient with down-beating nystagmus, one patient with up-beating nystagmus), and HSN was found in 11 patients (four patients with ipsilesional beating nystagmus, five patients with contralesional beating nystagmus, two patients with down-beating nystagmus). All 21 patients had normal results of gaze-evoked nystagmus, saccades, smooth pursuit, and optokinetic tests.

**Table 3:** Ipsilesional vestibulo-cochlear involvement in patients with unilateral ILPC on vHIT.

**Diagnosis**	**Caloric irrigation^a^: impaired horizontal canal (*n*, %)**	**oVEMPs^b^: impaired utriculus (*n*, %)**	**cVEMP^b^: impaired sacculus (*n*, %)**	**Impairment of any of the three: Calorics, oVEMP, cVEMP (*n*, %)**	**PTA (hearing loss^c^) (*n*, %)**	**Impairment of any of the four: Calorics, oVEMP, cVEMP, PTA (*n*, %)**
Vertigo/dizziness of unclear origin (*n* = 5)	2, 40%	0, 0%	0, 0%	2, 40%	1, 20%	3, 60%
Labyrinthine infarction (*n* = 2)	0, 0%	0, 0%	0, 0%	0, 0%	1, 50%	1, 50%
Vestibular migraine (*n* = 2)	1, 50%	0, 0%	0, 0%	1, 50%	0, 0%	1, 50%
Various causes^d^ (*n* = 4)	1, 25%	1, 33.3% (1 missing)	0, 0%(1 missing)	2, 50%	0, 0%	2, 50%
Total unilateral ILPC (*n* = 13)	4, 30.8%	1, 8.3% (1 missing)	0, 0% (1 missing)	5, 38.5%	2, 15.4%	7, 53.8%

a *Impairment of the horizontal canal was considered significant if the asymmetry ratio was >25% in favor of the other side*.

b *Impairment of vestibular-evoked myogenic potentials was considered significant if the asymmetry ratio was >30% in favor of the opposite side or if responses were bilaterally absent*.

c *Significant hearing-loss was defined as CPT-AMA values >20%*.

d *Various causes include:Vestibular neuritis (n = 1), Meniere disease (n = 1), Autoimmune inner ear disease (n = 1), Labyrinthine concussion (n = 1)*.

*LPC, loss of the posterior canal(s); vHIT, video-head-impulse test; oVEMP, ocular vestibular-evoked myogenic potentials; cVEMP, cervical vestibular-evoked myogenic potential; PTA, pure tone audiometry*.

Among two patients with BLPC, CP value of >25% was found in one patient with ILPC (right HSCC hypofunction), the other patient showed normal caloric test (**Table 5**). SN and HSN were found in all patients (down-beating nystagmus). And all patients had normal results of gaze-evoked nystagmus, saccades, smooth pursuit, and optokinetic tests.

### oVEMP and cVEMP Results

Among 21 patients with ULPC, two patients showed ipsilesionally reduced oVEMPs, one patient showed contralesionally reduced oVEMPs, 17 patients showed normal oVEMPs, and the remaining one patient did not undergo oVEMP test. While among two patients with BLPC, one patient showed unilaterally reduced oVEMPs, one patient showed normal oVEMPs.

Among 21 patients with ULPC, one patient showed ipsilesionally reduced cVEMPs, four patients showed contralesionally reduced cVEMPs, 15 patients showed normal oVEMPs, respectively, 1 patient did not undergo cVEMP test. Among two patients with BLPC, normal cVEMPs were observed in both patients. Rates of reduced oVEMP and cVEMP in patients with ULPC and BLPC who had different underlying diagnoses were shown in Tables 2–5.

**Table 4:** Ipsilesional vestibulo-cochlear involvement in patients with unilateral non-ILPC on vHIT.

**Diagnosis**	**vHIT: impaired horizontal canal (*n*, %)**	**Caloric irrigation^a^: impaired horizontal canal (*n*, %)**	**Impairment of any of the two: vHIT, Calorics (*n*, %)**	**oVEMPs^b^: impaired utriculus (*n*, %)**	**cVEMP^b^: impaired sacculus (*n*, %)**	**Impairment of any of the three: Calorics, oVEMP, cVEMP (*n*, %)**	**PTA (hearing loss^b^) (*n*, %)**	**Impairment of any of the four: Calorics, oVEMP, cVEMP, PTA (*n*, %)**
Vertigo/dizziness of unclear origin (*n* = 2)	2, 100%	2, 100%	2, 100%	1, 50%	1, 50%	2, 100%	0, 0%	2, 100%
Autoimmune inner ear disease (*n* = 2)	1, 50%	1, 50%	1, 50%	0, 0%	0, 0%	1, 50%	1, 50%	2, 100%
Various causes^d^ (*n* = 4)	4, 100%	1, 25%	4, 100%	0, 0%	0, 0%	2, 50%	1, 25%	1, 25%
Total unilateral LPC (*n* = 8)	7, 87.5%	4, 50%	7, 87.5%	1, 12.5%	1, 12.5%	4, 50%	2, 25%	5, 62.5%

a *Impairment of the horizontal canal was considered significant if the asymmetry ratio was >25% in favor of the other side*.

b *Impairment of vestibular-evoked myogenic potentials was considered significant if the asymmetry ratio was >30% in favor of the opposite side or if responses were bilaterally absent*.

c *Significant hearing-loss was defined as CPT-AMA values >20%*.

d *Various causes include: Labyrinthine infarction (n = 1), Otitis media (n = 1), Vestibular neuritis (n = 1), Intralabyrinthine hemorrhage (n = 1)*.

*LPC, loss of the posterior canal(s); vHIT, video-head-impulse test; oVEMP, ocular vestibular-evoked myogenic potentials; cVEMP, cervical vestibular-evoked myogenic potential; PTA, pure tone audiometry*.

**Table 5:** Vestibulo-cochlear test and underlying diagnoses in patients with ILPC on vHIT.

**ID/Sex/Age^a^**	**Main complaint^c^**	**vHIT^c^**		**SN^c^**	**HSN^c^**	**CP^b^**	**oVEMP^b^**	**cVEMP^b^**	**PTA^b^**	**Diagnosis**
		**aVOR gains**	**Compensatory saccades**							
1/M/50	D	RP↓	LP + RP	-	RB	-	-	-	L↓	Vestibular migraine
2/M/40	D+Unsteadiness	RP↓	RP	-	DB	-	-	-	R↓	Vertigo/dizziness of unclear origin
3/F/35	D	LP↓	LP	-	LB	L↓	-	-	-	Vestibular migraine
4/M/67	D	LP↓	LP + RP	-	-	L↓	ND	ND	(L+R) ↓	Vestibular neuritis
5/F/41	V	RP↓	RP	DB	DB	L↓	-	L↓	-	Autoimmune inner ear disease
6/F/65	V	RP↓	RP	-	RB	-	R↓	-	(L+R) ↓	Meniere disease
7/F/58	D	RP↓	LP + RP	LB	-	R↓	-	-	-	Vertigo/dizziness of unclear origin
8/F/65	D	RP↓	RP	-	-	-	-	-	-	Labyrinthine concussion
9/F/20	D	RP↓	RP	UB	RB+DB	-	-	-	-	Vertigo/dizziness of unclear origin
10/F/65	D	RP↓	RP	-	-	-	-	-	-	Labyrinthine infarction
11/M/56	D	RP↓	RP	-	LB	R↓	-	-	-	Vertigo/dizziness of unclear origin
12/F/78	V	RP↓	RP	-	-	-	-	L↓	-	Vertigo/dizziness of unclear origin
13/F/62	V	RP↓	RP	-	-	-	L↓	L↓	R↓	Labyrinthine infarction
14/M/82	D+ Unsteadiness	(LP + LH + RH) ↓	LP + RP	LB	RB	-	-	-	(L+R) ↓	Labyrinthine infarction
15/F/48	D+ Unsteadiness	(RP + RH) ↓	RP	LB	-	R↓	R↓	R↓	-	Vertigo/dizziness of unclear origin
16/F/68	D	(RP + RH) ↓	RP	-	-	-	-	-	(L+R) ↓	Vestibular neuritis
17/M/58	D	(RP + RH) ↓	RP	-	-	R↓	-	-	-	Vertigo/dizziness of unclear origin
18/F/60	D	(RP + RH) ↓	RP	-	-	R↓	-	L↓	(L+R) ↓	Autoimmune inner ear disease
19/M/44	V	(RP + RH) ↓	RP	-	LB	-	-	-	-	Otitis media
20/M/47	V+ Unsteadiness	(LP + LH) ↓	LP	LB	RB	L↓	-	-	L↓	Labyrinthine infarction
21/M/70	V	(RP + LH) ↓	RP	LB	LB	-	-	-	R↓	Autoimmune inner ear disease
22/F/72	D+Unsteadiness	(LP + RP) ↓	LP + RP	DB	DB	R↓	R↓	-	-	Vestibular paroxysmia
23/M/59	V	(LP + RP + LA + RH) ↓	RP	DB	DB	-	-	-	(L+R) ↓	Autoimmune inner ear disease

a *Patients with ID 1–13 are unilateral ILPC, patients with ID 14–21 are unilateral non-ILPC, patient with ID 22 is bilateral ILPC, patient with ID 23 is bilateral non-ILPC*.

b *Impairment of the horizontal canal was considered significant if the asymmetry ratio was >25% in favor of the other side. Impairment of vestibular-evoked myogenic potentials was considered significant if the asymmetry ratio was >30% in favor of the opposite side or if responses were bilaterally absent. Significant hearing-loss was defined as CPT-AMA values >20%*.

c *M, Male; F, Female; V, vertigo; D, non-vertigo dizziness; D+Unsteadiness, non-vertigo dizziness with unsteadiness; aVOR, angular vestibulo-ocular reflex; RP↓, right posterior semicircular canal hypofunction; LP↓, left posterior semicircular canal hypofunction; UB, Up-beating nystagmus; DB, Down -beating nystagmus; LB, Left-beating nystagmus; RB, Right -beating nystagmus; ”-”, no nystagmus/normal test results; ND, Not done; LPC, loss of the posterior canal(s); vHIT, video-head-impulse test; oVEMP, ocular vestibular-evoked myogenic potentials; cVEMP, cervical vestibular-evoked myogenic potential; PTA, pure tone audiometry; SN, spontaneous nystagmus; HSN, head-shaking nystagmus; CP, canal paresis value*.

### PTA Results

Among 21 patients with ULPC, hearing loss was identified in 10 (52.4%) patients. Of these 10 patients, four patients showed hearing loss ipsilesionally as ULPC, five patients showed bilateral hearing loss, one patient showed hearing loss on the opposite side as ULPC (Table 2). Among 13 patients with unilateral ILPC, hearing loss was identified in five patients, two patients showed hearing loss ipsilesionally as ILPC, two patients showed bilateral hearing loss, one patient showed hearing loss on the opposite side as ILPC (Table 3). Vestibulo-cochlear test results for patients with ILPC and non-ILPC were shown in Tables 2–5.

Among two patients with BLPC, bilateral hearing loss was noted in non-ILPC patient, normal PTA finding was observed in one patient with ILPC (Table 5).

## Discussion

Based on the video-head-impulse test, we detected vestibular function in 448 patients with dizziness/vertigo/unsteadiness, LPC was identified in 23 patients, among these patients, ILPC was noted in 14 patients, accounting for 3.1% of all patients studied, and ULPC was more common than BLPC, this result is similar to the previous findings of Tarnutzer et al. (2), suggesting that LPC is uncommon in patients with dizziness/vertigo, vHIT has important value in the diagnosis of LPC (1), which deserves further attention from clinicians.

### Clinical Characteristics and Etiology of ILPC

Among 14 patients with ILPC identified in this study, 92.9% (13/14) patients had unilateral ILPC, and only one patient had bilateral ILPC. In patients with unilateral ILPC, the rate of ipsilesional impairment on caloric irrigation, or cVEMP/oVEMP, or PTA was 53.8%. And the causes of ILPC were vertigo/dizziness of unclear origin, labyrinthine infarction, vestibular migraine and other diseases (Table 3). Murofushi et al. (26) reported that patients with vestibular migraine had lesions in the central nervous system or in the peripheral vestibular system, suggesting that the causes of ILPC can be peripheral origin or central origin. The etiologies of ILPC in our study are different from the findings of Tarnutzer et al. (2), they showed that the most frequent underlying diagnoses were Menière's disease, vestibular neuritis, and vestibular schwannoma. In this study, only one patient had Ménière's disease, the reason may be that all patients included in the study were from Vertigo center of Neurology department, they were admitted with chief complaint of vertigo and dizziness, most of patients with Menière's disease experienced hearing loss who visited out-patient clinic were not included.

We further compared the differences between patients with unilateral ILPC and unilateral non-ILPC (Table 5), and found that: (1) at the time of disease onset, dizziness/vertigo were more commonly seen in both patients with unilateral ILPC and unilateral non-ILPC (92.3 vs. 62.5 %, *p* > 0.05); (2) except vHIT, ipsilateral impairment of any of the tests (caloric irrigation, cVEMP, oVEMP, or PTA) was observed in both patients with unilateral ILPC and unilateral non-ILPC (53.8 vs. 62.5%, *p* > 0.05); (3) diagnoses were clear in most of patients with unilateral ILPC and unilateral non-ILPC (61.5 vs. 75%, *p* > 0.05). The results suggested that since it is difficult to distinguish between unilateral ILPC and unilateral non-ILPC based on the clinical characteristics of patients, vHIT is the only diagnostic test to distinguish between patients with unilateral ILPC and unilateral non-ILPC.

### Clinical Characteristics and Etiology of ULPC

At present, few studies have focused on the clinical characteristics and etiology of ULPC. We found that patients with ULPC most often presented with dizziness/vertigo (17/21, 81.0%), and few patients presented with unsteadiness (4/21, 19.0%). ULPC may be associated with atherosclerosis, inflammation and immunity: (1) in this study, in addition to about 1/3 of patients with vertigo/dizziness of unclear origin, atherosclerosis-associated factors were the main causes of LPC. The previous studies indicate that patients with labyrinthine infarction are often accompanied by atherosclerosis-related factors, and LPC can occur in patients with labyrinthine infarction. The reason may be that vascular lesions of the branch of internal auditory artery, i.e., the posterior vestibulocochlear artery, may affect the function of posterior canal ampulla and the inferior portion of the saccule (27, 28). The above findings are consistent with results of our study showing that the occurrence of LPC may be closely related to atherosclerosis-related risk factors; (2) in addition to possible atherosclerosis-related factors, we found that inflammation can also invade the inferior vestibular nerve and cause ULPC, the cause of ULPC was vestibular neuritis in 9.5% (2/21) patients. Vestibular neuritis mainly affects the vestibular nerve and superior portion of the vestibular labyrinth, and the inferior vestibular nerve is less affected (29). Our result is consistent with the findings of a study by Kim et al. (30), showing that the occurrence of ULPC may be related to inferior vestibular neuritis; (3) in this study, we found that the diagnosis was autoimmune inner ear disease in four patients with LPC, ULPC was noted in three out of these four patients, suggesting that autoimmune inner ear disease may also cause ULPC. Das et al. (16) showed that the typical clinical presentation of autoimmune inner ear diseases is progressive fluctuating hearing loss, both ears and one ear can be involved, and peripheral cochlear function can be affected, but the study did not use vHIT to further investigate the function of the involved vestibular organs and semicircular canals. Our results suggest that the occurrence of LPC may be related to autoimmune diseases.

**Figure 3:**
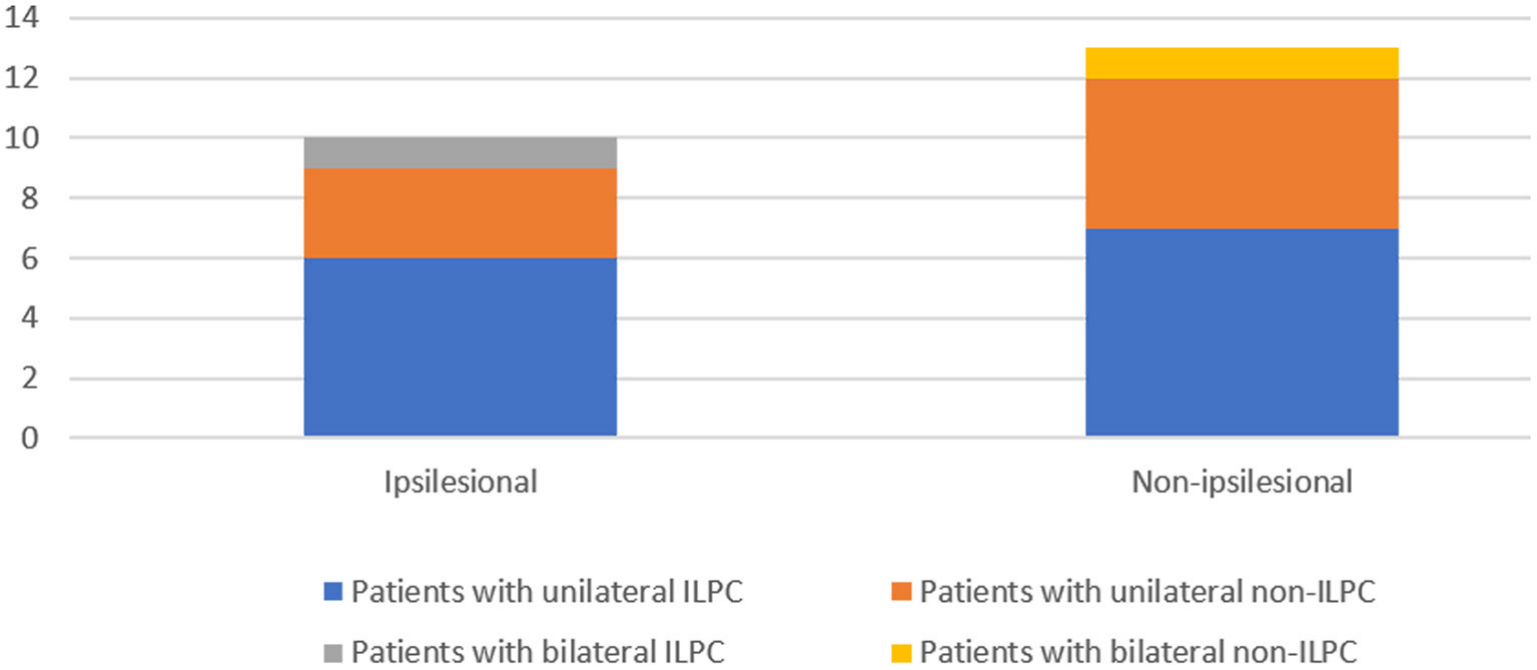
Impairment of any of the four: Calorics, oVEMP, cVEMP, and PTA in patients with the loss of the posterior canal (s) (LPC).

In this study, results of vestibulo-cochlear tests in patients with ULPC showed that except vHIT, ipsilateral impairment of any of the other vestibular-cochlear tests (caloric test, cVEMP, oVEMP, and PTA) was observed in 57.1% (12/21) patients with ULPC (Figure 3). This result is similar to the findings of previous study by Tarnutzer et al. (2), indicating that ULPC is mostly accompanied by vestibulo-cochlear impairment on multiple tests, and there is no significant difference between unilateral ILPC and unilateral non-ILPC (LPC+LHC). In this study, the number of patients who showed normal cVEMPs was high, we speculated that this may be due to that patients were in mild condition and non-acute phase, most patients (78.3%) had disease duration of > 2 weeks, in addition, we only performed air-conduction cVEMP testing in patients, and did not perform bone-conduction VEMP testing, this may also cause low incidence of cVEMPs abnormality.

### Clinical Characteristics of BLPC

In this study, we identified 2 (2/23, 8.7%) patients with BLPC. Dizziness with unsteadiness and vertigo are the main complaints of the patients with BLPC. Our results suggest that the occurrence of BLPC may be associated with autoimmune diseases and vascular factors, which can affect bilateral inferior vestibular nerve, causing down beating spontaneous nystagmus, and down beating head-shaking nystagmus (31). Although the HSCC function detected by both vHIT and caloric tests did not meet the diagnostic criteria for bilateral vestibular vestibulopathy (BVP), however, these patients had a vHIT gain of <0.70 for bilateral PSCC, with main complaints of vertigo/dizziness and unsteadiness, which can be used as complementary methods for diagnosing BVP. With the application of new evaluation methods, future research will further refine and improve the diagnostic criteria for subtypes of BVP.

### Study Limitations

LPC are relatively rare in clinical practice, so the sample size of the study is small. Further studies with larger sample size are needed to confirm the results. In addition, this study only included patients admitted to our hospital, patients attending outpatient clinics are needed to be included in future studies.

## Conclusion

vHIT is a fast and effective method for assessing LPC, which can be used to detect isolated PC dysfunction. The causes of ILPC were peripheral origin or central origin. Patients with ILPC and ULPC mostly presented with dizziness/vertigo, and ULPC was often accompanied by ipsilateral vestibulo-cochlear impairment.

## Data Availability Statement

The original contributions presented in the study are included in the article/supplementary material, further inquiries can be directed to the corresponding author/s.

## Ethics Statement

The studies involving human participants were reviewed and approved by the ethics committee of Aerospace Center Hospital, Peking University Aerospace School of Clinical Medicine, Beijing, China. The patients/participants provided their written informed consent to participate in this study.

## Author Contributions

XY: conception and design of the work. ZL: writing the manuscript. ZL, BL, and HS: acquisition of data for the work. KL, BS, XLi, and XLin: analysis and interpretation of data for the work. All authors contributed to the article and approved the submitted version.

## Conflict of Interest

The authors declare that the research was conducted in the absence of any commercial or financial relationships that could be construed as a potential conflict of interest.
